# Integrating thermodynamics and molecular kinetics into western blotting teaching

**DOI:** 10.3389/fmolb.2025.1640740

**Published:** 2025-08-14

**Authors:** Zhiyong Wang, Min Wang, Yan Yu, Yanxin Lu, Qiang Xia, Pei Wei

**Affiliations:** ^1^ Department of Immunology, Zhuhai Campus of Zunyi Medical University, Zhuhai, China; ^2^ Department of Pharmaceutics, Zhuhai Campus of Zunyi Medical University, Zhuhai, China

**Keywords:** Western blotting, antigen (Ag), antibody (Ab), thermodynamics, kinetics

## Introduction

The Western blotting technique, which was first detailed by Towbin et al. (1979), is mainly used for the identification of proteins from complex biological samples. It sensitively detects and quantifies specific proteins from fairly complex biological mixtures, offering a great potential for new discoveries in the field of life sciences (Sule et al., 2023; Meftahi et al., 2021; Begum et al., 2022). Even in basic laboratories, immunodetection is a challenging phase, which can result in extremely weak or no signals. In addition, enhanced background, non-specific bands, or uneven banding of the samples can be commonly observed, leading to false positive or negative results (Liu et al., 2014; Butler et al., 2019). In traditional modes of teaching, these problems are assumed to be solved through the empirical adjustment of the experimental conditions. These adjustments include modification of antibody concentration, incubation time, or blocking reagents without strongly linking various causes to unifying the conceptual framework for students. Much reliance on experimental trial-and-error instead of systematic hypothesis testing leads to such problems.

Herein, we introduced thermodynamic concepts and principles of molecular thermal motion to provide a robust and unified framework for Western blotting. The thermodynamics of antigen-antibody interaction are governed by Gibbs free energy (Δ*G*), which is a combined contribution of enthalpy change (Δ*H*) and entropy change (Δ*S*) (Sagawa et al., 2003; Tamil et al., 2002). Kinetically, these interactions are controlled by the association rate constant (k_on_) and the dissociation rate constant (k_off_) (Mason and Williams, 1980; Rossini and Dimitrov, 2021). By mapping experimental variables, such as buffer systems, temperature, antibody concentration, and incubation time onto these biophysical parameters (Rossini and Dimitrov, 2021; Voets, 2017), students can predict the effect of each adjustment on equilibrium occupancy and kinetic rates and attribute common troubleshooting issues to suboptimal thermodynamic or kinetic conditions. This article first introduces the theoretical basis of antigen-antibody binding and then elucidates how suboptimal conditions can lead to common problems when running Western blotting. Finally, the article proposes teaching design strategies based on this framework.

In accordance with contemporary evidence-based STEM teaching practices, students must apply fundamental principles of the subject to actual predictions of outcome and diagnosis of failure. Students transition from being passive recipients of a protocol to active participants in the intellectual process. This active learning principle supports “scientific reasoning” by having students formulate hypotheses and devise logical troubleshooting strategies that are grounded in theory. By doing so, students move beyond procedural “hands-on learning” to a deeper “minds-on” realization of the actual scientific method.

## A biophysical primer: key concepts for immunodetection

A solid foundation in biophysical principles governing molecular interactions is needed to evaluate how experimental variables affect Western blotting. The binding between an antibody and its target antigen is a dynamic equilibrium rather than a static event. It is described by laws of thermodynamics and molecular kinetics. We introduce these basic principles in an intuitive way.

**FIGURE 1:**
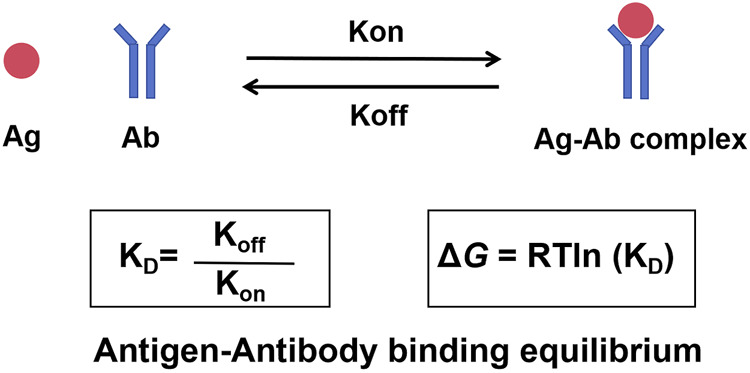
The dynamic equilibrium of antigen-antibody binding. The interaction between an antibody and its antigen is a reversible process governed by kinetic and thermodynamic principles. The association rate constant (k_on_) describes the speed at which the antibody binds to the antigen to form a complex. The dissociation rate constant (k_off_) describes the speed at which the complex breaks apart. The ratio of these two rates defines the dissociation constant (K_D_), a measure of binding affinity. A low K_D_ signifies high affinity. The spontaneity of this binding is determined by the Gibbs free energy change (Δ*G*), which is directly related to K_D_.

Kinetic rate constants are at the center of the interactions (Figure 1). Imagine antibody and antigen molecules to be constantly moving dancers in a crowded ballroom. The association rate constant (k_on_) is the rate at which molecules find each other and pair. This association depends on concentration (more dancers equate to more pairs) and how quickly they can diffuse. The rate at which an existing pair separates is the dissociation rate constant (k_off_)—an intrinsic property of how compatible a pair is. As such, a strong couple will separate slowly (low k_off_) but a weak couple will separate quickly (high k_off_). The overall strength (or “stickiness”) of this interaction is represented as the dissociation constant (K_D_), or the ratio of the two rates: K_D_ = k_off_/k_on_. A small K_D_ value means strong affinity and a stable, long-lived complex (for example, k_off_ is very slow or k_on_ is very fast, or both). On the contrary, a big K_D_ signifies weak affinity and therefore transient interaction. Usually, one uses an antibody with a very low K_D_ for the target antigen but a very high K_D_ for other membrane proteins.

Gibbs free energy change (Δ*G*) drives this spontaneous binding. Δ*G*, the net change of a reaction in terms of energy, determines whether a reaction proceeds. Affinity relates directly to free energy—Δ*G* = RTln (K_D_)—where R is the gas constant and T is absolute temperature. A small K_D_ (high affinity) relates to very negative Δ*G* (keeping in mind that binding will occur thermodynamically favorably and spontaneously). Positive or about zero Δ*G*s suggest the binding is unfavorable and will not occur significantly.

**FIGURE 2:**
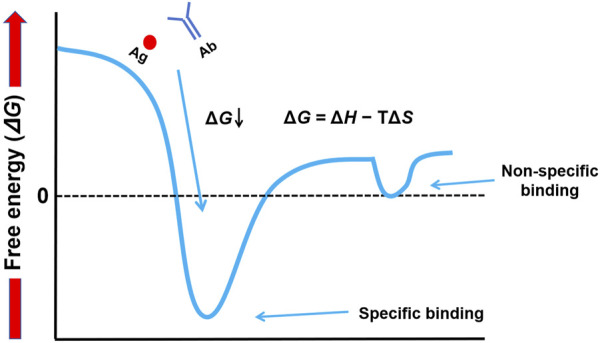
Conceptual energy landscape of molecular interactions in Western blotting. This diagram illustrates the thermodynamic difference between specific and non-specific binding. Specific binding is characterized by a deep energy well (highly negative Δ*G*), indicating a stable, high-affinity interaction. In contrast, non-specific interactions are represented by shallow energy troughs (Δ*G* near zero), which are unstable and easily disrupted by washing.

Gibbs free energy is a measure of change in two components—enthalpy (Δ*H*) and entropy (Δ*S*)—that are linked by the equation Δ*G* = Δ*H* − TΔ*S*. Δ*H* represents the heat of binding and is caused by energy released from forming favorable noncovalent bonds such as hydrogen bonds, electrostatic interactions, and/or van der-Waals forces. A negative Δ*H* contributes favorably to binding. The measure of disorder is entropy (Δ*S*). The binding of two molecules into one complex reduces their freedom and is hence entropically unfavorable; an entropic gain would often be more from the release of ordered water molecules at the binding interface. The intricate play between enthalpy and entropy determines Δ*G*. This distinction, which contrasts high-affinity specific binding (very negative Δ*G*, deep energy well) and low-affinity nonspecific interactions (Δ*G* near zero, shallow energy troughs), forms the crux of mastering Western blotting. The difference in conceptual orientation is captured in our energy landscape diagram (Figure 2), which serves throughout the article to clarify how experimental conditions can be manipulated to achieve the selective promotion of the former while minimizing such.

### Challenges in teaching western blotting

Immunodetection in Western blotting involves incubation with antibodies, washing steps, and signal detection and consists of a series of extremely sensitive biomolecular interactions. The successful results of an experiment rely on the regulation of these events, which can increase the specificity of the obtained signals and decrease background noise (Towbin et al., 1979). However, in a teaching setting, it usually leads to conceptual misunderstanding among students, leading to their frustration (Tu and Levin, 2022). They usually experience such challenges because they cannot identify a logical explanation for common conditions, such as no signal or weak signal, enhanced background, or non-specific bands (Towbin et al., 1979; Kroon et al., 2022). Problems in antibody affinity, concentration, incubation kinetics, or procedural errors are assumed to be the main culprit (Gilda et al., 2015).

Teaching methodologies suffer from time or resource limitations. They concentrate too heavily on the “how-to” of Western blotting and pay less attention to “why-it-works.” Laboratory procedures should be explained in detail. A superficial learning can be achieved when students heavily rely on doing steps without thinking about the principles. Students never develop the analytical skills needed to address new or complex problems when troubleshooting is presented only in the form of a list of recognized problems and their solutions (Tu and Levin, 2022). Real experimental skills require analyzing a problem, hypothesizing on the basis of science, and logically designing steps for troubleshooting.

### A unified conceptual framework: thermodynamics and molecular thermal motion in western blotting

Based on the concepts of thermodynamics and theories of molecular thermal motion, we propose an instructional framework to move away from procedural learning and empower students to achieve stronger troubleshooting skills. Although these principles support all chemical and biochemical reactions, they are rarely used in most practical demonstrations of techniques, such as Western blotting.

### Understanding antigen-antibody binding based on thermodynamics and molecular thermal motion

Antigen-antibody binding is an extremely transient and dynamic process characterized by the formation and breakage of molecules via chance encounters (García-Sánchez et al., 2023). The chances of such encounters are determined by the Brownian motion of antibody molecules, which are constantly agitated in their liquid environment. The rate of diffusion and collision of antibodies is directly linked to temperature increase. Antigen-antibody binding may be a reversible non-covalent interaction. Their binding strength, or affinity, is expressed as the dissociation constant (K_D_, wherein lower values imply stronger affinity) and as its reciprocal, the association constant (K_A_, wherein higher values imply stronger affinity) (Mason and Williams, 1980). Both equilibrium constants are linked to the ratio between two rate constants, one for association (k_on_), which describes the bonding speed, and one for dissociation (k_off_), which describes the unbinding speed (K_D_ = k_off_/k_on_) (Mason and Williams, 1980; Rossini and Dimitrov, 2021). The concentration of antigen-antibody binding affinity is correlated with the Gibbs’ free energy change (Δ*G*) of the binding reaction (Sagawa et al., 2003). The change in Gibbs free energy is defined as Δ*G* = Δ*H*−TΔ*S*. It indicates the spontaneity of an antigen-antibody (Ag-Ab) binding reaction and its position at equilibrium, with more negative values suggesting stronger binding and stable complexes. This binding strength is also affected by the following variables: enthalpy changes (Δ*H*), which represents the net energy change occurring during the formation of non-covalent bonds, including hydrophobic interactions, hydrogen bonds, electrostatic forces, and van der Waals forces; and changes in entropy (Δ*S*), which is explicitly associated with solvent reorganization and changes in molecular conformational freedom. Translation or rotational entropy is inhibited in the case of Ag-Ab complexes, while the release of water molecules from the interface during hydrophobic interactions can increase solvent entropy, which often increases the net positive Δ*S* (Sagawa et al., 2003; Schwarz et al., 1995).

### Interpreting common experimental variables of western blotting through the new framework

By mastering these core principles, students can understand immunodetection steps as a series of molecular interactions governed thermodynamically and kinetically (Table 1). Theoretically, this should maximize signal-to-noise ratios. In other words, high-affinity specific Ag-Ab binding (highly negative Δ*G*) should be thermodynamically favorable, but interactions of antibodies with other proteins or the membrane itself are thermodynamically unfavorable and result in positive or negligibly negative Δ*G*).

**TABLE 1:** Biophysical interpretation for Western blotting variables.

Variable	Biophysical rationale	Optimal outcome	Common problems and their biophysical causes
Temperature	Affects kinetics (k_on_/k_off_), *TΔS* in *ΔG*	Accelerates k_on_ to equilibrium without denaturation or high k_off_	Too high: Protein denaturation (unfavorable *ΔG*); high koff causes signal loss Too low: Slow kinetics (low k_on_); equilibrium not reached
Antibody Conc	Mass action: higher concentration drives binding	Balances specific signal vs. non-specific binding	Too high: High background from non-specific binding; antibody aggregation Too low: Weak/no signal from insufficient binding
Agitation	Enhances mass transport, increasing apparent k_on_	Uniform, faster binding for a consistent signal	Insufficient: Slow diffusion (low apparent k_on_) causes weak or uneven signals Too vigorous: Shear forces increase k_off_ of weak interactions, causing signal loss
Blocking	Makes non-specific binding *ΔG* unfavorable by occupying sites	Inert surface maximizes signal-to-noise by preventing background	Incomplete: Exposed sites cause non-specific binding (high background) Excessive: Masks antigen, reducing k_on_ (weak signal)
Washing	Removes non-specifics by leveraging their high k_off_	Removes non-specific antibodies, reducing background	Too gentle: Fails to remove non-specifics (high background) Too harsh: Dissociates specific complexes (weak signal)
pH	Alters protein charge and conformation, affecting *ΔH* and *ΔS*	Optimal conformation and charge for most favorable *ΔG* (high affinity)	Deviation (too high or low): Reduces affinity (less negative *ΔG*); can cause denaturation or non-specific adsorption (high background)
Ionic Strength	Screens charges and modulates hydrophobic interactions, impacting *ΔH* and *ΔS*	Suppresses non-specific electrostatic binding while preserving specific binding	Too high: Weakens specific electrostatic binding (signal loss); increases non-specific hydrophobic binding Too low: Fails to screen charges; protein aggregation

### Temperature

Undoubtedly, temperature affects the speed and equilibrium of antigen-antibody binding (Johnstone et al., 1990). In terms of molecular thermal motion, increasing the temperature enhances the Brownian motion of antibody molecules, thereby increasing their collision frequency with the antigen and elevating the constant for the association rate (k_on_) (Johnstone et al., 1990; Levison et al., 1968). Compared to room temperature or 4 °C, higher temperatures (for example, 37 °C) increase the rate of formation and attainment of the equilibrium in this antigen-antibody complex (Johnstone et al., 1990; Stan et al., 2019). However, too high temperatures may lead to adverse results. First, a high temperature may denature antibodies or antigens, disrupt the binding site conformation, and lead to a greater reduction in affinity. Second, since antigen-antibody binding is a reversible reaction, higher temperatures will increase the dissociation rate constant (Johnstone et al., 1990; Wakayama and Sugiyama, 2012). Based on Gibbs free energy change Δ*G* = Δ*H*−TΔ*S* (Voets, 2017), when the enthalpy change (Δ*H*) of binding reaction remains less sensitive to temperature, Δ*G* should increase or be positive using sufficiently high temperatures, preventing complex formation and inducing the dissociation of formed complexes. Hence, the temperature for Western blotting should provide a balance between kinetic acceleration and thermodynamic stability (as well as biomolecular activity) (Johnstone et al., 1990; Stan et al., 2019).

### Antibody concentration

Antibody concentration determines how fast and how much antigen-antibody binding occurs. According to the law of mass action, the increase in antibody concentration can enhance the probability of collision of antibody molecules with antigens, thereby increasing the association rate constant kon, accelerating complex formation, and favoring the equilibrium toward complex formation (Koch et al., 2018). This definitely leads to poor detection of low-abundance antigens after a short length of incubation. Extremely high antibody concentrations may allow for more than simple binding to the target antigen and lead to a high collision rate with many non-specific sites on the membrane. In samples with weak binding capabilities (these sites have larger K_D_ values, corresponding to less negative Δ*G*), this non-specific binding will result in drastically greater cumulative collision numbers at significantly larger K_D_ values. These non-specific reactions can increase the background signal, which can overshadow the specific signal (Algenäs et al., 2014; Loeffler and Klaver, 2017). Collectively, high concentrations of antibodies may promote the aggregation of antibodies, and aggregated antibodies prefer non-specific adsorption (Perchiacca and Tessier, 2012).

### Agitation

There appears a relatively stagnant liquid layer on the membrane surface during static incubation requiring antibodies to traverse this layer via slow Brownian movement (diffusion) to reach immobilized antigens (Nygren and Stenberg, 1985; Pereiro et al., 2020). Broad agitation generates macroscopic convection that effectively thins this stagnant layer, thereby accelerating the migration of antibodies toward the membrane surface and ensuring a more uniform distribution of antibodies throughout the incubation system (Pereiro et al., 2020; Rathaur et al., 2020). This increases the chance of effective collision frequency between antibodies and antigens, thereby increasing the apparent association rate constant (k_on_) and contributing to a more rapid and uniform binding (Pereiro et al., 2020; Hu et al., 2007). Overzealous or inappropriate agitation methods, such as those generating excessive foam or vortices, can exert real forces of mechanical shear on antigen-antibody complexes with weaker bindings (greater K_D_, less negative Δ*G* or relatively greater k_off_). This may increase energy and loosen feeder lines that produce some bound-specific antibodies and decrease outputs (Shieh and Patel, 2015).

### Blocking

The purpose of blocking is to enhance the specificity of subsequent immunodetection by occupying non-specific binding sites on the membrane (Kothari and Mathews, 2015). Ideal blocking agents, such as BSA or skim milk powder, effectively bind to vacant areas on PVDF or NC membranes that may otherwise engage in hydrophobic or electrostatic interactions with antibodies (Kothari and Mathews, 2015). This forms an inert protein layer, which makes Δ*G* for non-specific interactions between antibodies and the blocked regions highly unfavorable. In another word, the K_D_ value for these sites becomes extremely large for the detecting antibody, thereby significantly reducing background interference (Schwartz and Bochkariov, 2017). This beneficial blocking effect originates from the strong interaction of blocking molecules with the membrane surface (negative Δ*G*), and the “repulsive” or at least “neutral” surface characteristics of the blocking layer for antibodies.

However, excessive blocking (e.g., excessively high concentration of blocking agents, excessively long blocking time, or inherent unexpected properties of the blocking agents) can lead to unfavorable outcomes (Kothari and Mathews, 2015). Blocking agents with extremely high concentration or excessively large size may not merely remain on ‘blank’ regions. Through non-specific adsorption or intermolecular stacking, such blocking agents may partly or even completely mask or encapsulate target antigens already immobilized on the membrane. This physical masking significantly increases the steric hindrance for the primary antibody, which should bind to the target antigen epitope, thereby increasing the activation energy needed for specific antigen-antibody binding. This can render an inherently favorable binding Δ*G* (e.g., a highly negative Δ*G* for specific binding). It can be less favorable due to the need to overcome an additional energy barrier, or can be kinetically very slow (significantly reduced k_on_). Blocking agents with weak and non-specific attractive forces with the target antigen can transiently or weakly bind to the target antigen in the presence of excessive blocking. These weak interactions may perturb the optimal binding conformation between the primary antibody and the target antigen or partly occupy certain regions of the antigen epitope. This can increase the effective K_D_ value for the primary antibody binding to the target antigen (reduced affinity), with a less negative Δ*G* and a diminished specific signal. Excessively high concentrations of blocking agents (especially certain proteins like casein) can form aggregates under specific conditions. Due to their physicochemical properties (e.g., hydrophobicity, charge), these aggregates may non-specifically deposit on the membrane, potentially encapsulating some antibodies and introducing new background signals or interfering with the uniform diffusion of subsequent reagents.

### Washing

The specific and high-affinity binding of antigen to the antibody is characterized by a high negative Δ*G* value with a low K_D_ value, forming very stable complexes. On the contrary, low-specific or non-specific binding is characterized by a less negative Δ*G* (possibly near zero or slightly positive) along with a higher K_D_ and a relatively fast k_off_ rate, leading to the formation of unstable complexes that dissociate rapidly. Sufficient washing with the continuous flow of fresh wash buffer removes free antibodies from the solution. Based on Le Chatelier’s principle, washing removes loosely bound non-specific molecules that are in rapid association-dissociation dynamic equilibrium (Yukhananov et al., 2022). Detergents in the wash buffer, such as Tween-20, effectively reduce the strength of non-specific hydrophobic interactions by altering their Δ*H* and Δ*S*, thus rendering their binding Δ*G* less favorable. Therefore, detergents promote their dissociation from the immobilized target (Yukhananov et al., 2022). Such actions reduce the background signal, thus emphasizing a signal generated specifically by stable complexes and enhancing the signal-to-noise ratio and specificity of detection (Yukhananov et al., 2022; Norris et al., 2005).

However, some specific signals will be lost in case of too harsh or prolonged wash. In specific binding, when the affinity is not very high (e.g., K_D_ is in the intermediate range, Δ*G* is not significantly negative or k_off_ is small but not negligible), prolonged or high-stringency washing (e.g., in the presence of high detergent concentration, high ionic strength, and temperature) may dissociate the specific complexes and reduce the target signal. Such situations tend to be more evident in the detection of low-abundance target proteins or antibodies with moderate affinity.

### pH value

pH alters the ionization state and conformation of protein molecules (antigens, antibodies, and blocking agents) (Devanaboyina et al., 2013). The binding Δ*H* (electrostatic interactions and hydrogen bonds) and Δ*S* (conformational entropy and solvation entropy) are directly associated with the effects of pH. Each protein has an isoelectric point, which relies on pH and controls the protonation state of amino acid residues on the protein surface (e.g., lysine, arginine, glutamate, aspartate, and histidine), thereby determining the net charge and local charge distribution of proteins (Devanaboyina et al., 2013; Endo et al., 1987). Antigen-antibody interactions usually occur based on specific charge complementarity (electrostatic interaction favors, hence making Δ*H* more negative) and an extensive hydrogen bond network. An optimal pH (usually close to physiological pH 7.0–7.4) can maintain the epitope of the antigen and the CDRs of the antibody in a state of ionization and configuration that is most favorable for interaction and can increase the binding affinity (i.e., most negative Δ*G*, least K_D_) (Devanaboyina et al., 2013; Igawa et al., 2010). An appropriate pH is essential for preserving the structural integrity and biological activity of antibodies, especially enzyme-conjugated secondary antibodies, while an extreme pH value can prevent their denaturation and inactivation.

Buffer pH out of the optimal range for a given pair of antigen-antibodies can lead to various adverse situations. First, any alterations in the surface charge of proteins can interfere with existing electrostatic attractions or form electrostatic repulsions, weaken hydrogen bonding, or even induce conformational changes in important residues in the antigen epitope or in the CDRs of the antibody (Devanaboyina et al., 2013; Endo et al., 1987). All of these changes make Δ*H* and Δ*S* less favorable for specific binding (e.g., changes in conformational entropy), thus leading to a less negative or even positive Δ*G* and reducing affinity (increase in K_D_) or signal (Igawa et al., 2010). Secondly, acidic or alkaline pH values can lead to irreversible protein denaturation and a total loss of binding. Besides, any change in pH will alter the surface charge on the membrane or blocking agents, which will then unintentionally increase certain non-specific electrostatic adsorptions and result in a high background (Ruiz et al., 2019). For example, when the membrane is negatively charged and the antibody is positively charged, the Δ*G* can be favorable for non-specific adsorption via electrostatics.

### Ionic strength

The ionic strength is the primary modulator of electrostatic and hydrophobic interactions between proteins, which screens the charges and the hydration layers, thereby modulating the binding Δ*H* and Δ*S* (Sah et al., 2010). Thus, in the buffer, the salt ions Na^+^ and Cl^−^ (such as from NaCl) can effectively screen the electrostatic charges on the protein surface (Kamata et al., 1996). A modest increase in ionic strength reduces these long-range electrostatic forces via the salt-screening effect for background binding, which is mainly mediated by non-specific electrostatic attraction between antibodies and oppositely charged membrane surfaces or contaminating proteins (Kamata et al., 1996; Pisetsky et al., 2021). A modest increase in ionic strength will also decrease background non-specificity, leading to a less favorable Δ*H* contribution to Δ*G* for such non-specific interactions. In addition, certain ionic strength levels are also needed for protein native conformation and solubility, a factor also contributing to the stability of antibodies (Pindrus et al., 2018).

On the other hand, too high ionic strength can weaken not only non-specific electrostatic interactions but also electrostatic attractions or perfect salt bridge structures that bear on specific antigen-antibody binding (Kamata et al., 1996; Roy et al., 1999). If specific binding mainly relies on electrostatic interactions that yield a positive Δ*H*, a very high ionic strength will reduce that contribution, yielding a less negative Δ*G*, less binding energy (greater K_D_), and less signal (Roy et al., 1999). Typically, high salt concentrations (the “salting-out effect”) strengthen hydrophobic interactions. An ionic salt competes with water for hydration, forcing non-polar surfaces to aggregate and minimize their contact area with water, thereby producing greater solvent entropy Δ*S*, and more negative Δ*G* (Kumar et al., 2011). In some cases, this may accentuate specific hydrophobic binding but also augment non-specific hydrophobic adsorption, leading to increased background (Thompson et al., 2016). Very low ionic strength, on the other hand, not only screens off non-specific electrostatic interactions but also decreases the solubility or aggregation of certain proteins (Pindrus et al., 2018; Liu et al., 2023).

## Explaining common experimental issues of western blotting from a thermodynamic and kinetic perspective

### No signal or weak signal

A lack of signal or signal intensity observed in Western blotting indicates that the formation of a measurable amount of stable specific antigen-antibody complexes was somehow impaired or the formed complexes did not efficiently convert into a measurable signal (García-Sánchez et al., 2023). Thermodynamically, Western blotting with no signal usually signifies either that the Δ*G* for specific binding is not negative enough (i.e., low affinity and high K_D_ value) or is positive, suggesting that binding is non-spontaneous. Such apparent interactions may be affected by inadequate antibody concentration (inactivation), degradation of the target antigen, or masking of the epitopic sites, hence preventing precise non-covalent interactions (contributing a favorable Δ*H*) and conformational complementarity (contributing an unfavorable Δ*S*) (García-Sánchez et al., 2023; Liu et al., 2023). Translating into kinetics, a too-slow association rate constant (k_on_) owing to excessively low antibody concentration, a short incubation time, or a low temperature that inhibits molecular thermal motion and reduces the collision frequency of antibodies with target proteins, or hindered diffusion and excessive dissociation rate constant (k_off_) may prevent complex formation (Liu et al., 2023). Finally, enzyme inactivation and substrate failure in the downstream detection system can also prevent signal generation (Zhou et al., 2021).

### High background

High backgrounds in Western blotting primarily arise from the binding of a detecting antibody to a large area of non-target molecules in the membrane, or even to the membrane itself (Grainger et al., 2014). These non-specific adsorption events correspond to negative Δ*G*, but most time, not as favorable as specific binding. Such events are mostly caused by low or inadequate blocking, leaving many hydrophobic or charged surfaces on the membrane exposed, which can then specifically bind to antibody molecules through hydrophobic effects (yielding favorable Δ*S* and/or Δ*H*) or electrostatic attraction (offering favorable Δ*H*) for non-specific binding (García-Sánchez et al., 2023; Grainger et al., 2014). Extremely high concentrations of an antibody will increase the collisional frequency among species, thereby leading to the accumulation of small-affinity (large K_D_, less negative Δ*G*) non-specific interactions. Poor washing does not take full advantage of the larger k_off_ values of non-specific bindings to achieve their release through thermal motion and concentration gradients (García-Sánchez et al., 2023).

### Non-specific bands

The presence of so-called non-specific bands indicates that the primary antibody (or, indirectly, the secondary antibody) has established an affinity-driven binding with other proteins in the sample, with a different molecule compared to the target protein (Nakashima et al., 2019). These “erroneous” binding events are associated with a negative Δ*G*, although usually with a K_D_ larger than the antibody’s binding to the primary target. It primarily accounts for antibody cross-reactivity that recognizes similar-epitope non-target proteins. Specifically, structural similarity allows the formation of partially favorable non-covalent bonds, with Δ*H* and Δ*S* contributions leading to negative Δ*G* (Perween et al., 2021). Similarly, degraded products of the target protein provide their epitopes for antibody recognition, forming additional bands (Nakashima et al., 2019). Increased concentration of the antibody beyond certain limits can increase the chance of binding to these degraded products with lesser affinities. Both incomplete blockade or too gentle washing may allow cross-reactive binding events, whose strength is between strong specific binding and very weak non-specific adsorption (Nakashima et al., 2019).

### Uneven bands and poor reproducibility

Miscalibrated conditions or discrepancies in the experiments lead to the emergence of uneven bands or irreproducible results in Western blotting (García-Sánchez et al., 2023; Perween et al., 2021). Therefore, there are many uncontrolled conditions, such as thermal motion, diffusion rates, kinetic reaction rates, and even chemical thermodynamics. For example, differences caused by poor electrophoresis or transfer of proteins (gel defects, air bubbles, and uneven pressure) can occur in the initial distribution of the antigen (Mishra et al., 2017; Garić et al., 2023). Insufficient washing or drying of the membrane directly affects the incidence frequencies and concentrations in the local context of collision. Non-exact control or fluctuation in temperature, pH, and ionic strength directly affects energies (Δ*H*, Δ*S*, and Δ*G*) and rates of all molecular associations, which results in differences in binding efficiency or final signal intensity (Mishra et al., 2017). Small technical changes can lead to noticeable differences in results via variations in molecular thermal motion, diffusion rates, chemical reaction kinetics, and molecular interaction.

### Troubleshooting based on the new framework

Traditional troubleshooting in Western blotting often relies on empirical “trial-and-error” methods. However, our proposed framework, based on thermodynamics and molecular thermal motion, aims to promote troubleshooting from an “empirical mode” to a “principle-driven mode.” The core diagnostic premise is that any issue in Western blotting, whether related to signal intensity or specificity, can be attributed to an imbalance in thermodynamic parameters (favorability of Δ*G*, reflecting affinity K_D_) and kinetic parameters (relative magnitudes of association rate constant kon and dissociation rate constant k_off_ of the interactions between target molecules and non-target molecules. Troubleshooting thus necessitates adjusting experimental conditions to achieve the favorable Δ*G* and appropriate kinetic rates for specific binding, while simultaneously minimizing the Δ*G* for non-specific binding or accelerating its dissociation.

### Teaching application example: coexistence of weak signal and high background

To provide an example of how this framework can help teaching, we analyze a scenario that commonly occurs. A student reports a Western blotting where the target band is very weak or absent but where there is generally a very high background over the whole membrane. An instructor expert in thermodynamics and molecular thermal motion can guide the students’ thinking:

Teacher says: “Let’s analyze these results. We have two primary issues: weak specific signal and high non-specific noise. From the framework we have studied, what might a weak specific signal imply thermodynamically and kinetically about antigen-antibody interaction?”

Student (guided): “This could suggest that ΔG associated with specific binding is not sufficiently negative, suggesting low affinity (high KD value), which can lead to the formation of very few stable complexes. Alternatively, the antibody concentration can be too low, with a reasonable KD, where mass action law suggests insufficient Ag-Ab complexes. Kinetically, perhaps the incubation time was not enough, and the antibody molecules did not have enough effective collisions with antigen via Brownian motion. Therefore, the reaction never attained equilibrium (kon-related events were simply too little), or the incubation temperature was too low, which decreased molecular motion rate and made kon too small.”

Teacher: “Excellent. Now, what about the high background? How does our framework explain antibodies non-specifically adsorbing across the membrane?”

Student (guided): “The binding of antibodies to non-specific sites on the membrane might be favorable thermodynamically (negative Δ*G*), or at least not sufficiently unfavorable (Δ*G* close to zero). Probably, our blocking step did not effectively provide enough energy barrier for subsequent antibody adsorption to these non-specific sites (i.e., it failed to make the Δ*G* for these non-specific interactions more positive or significantly less negative than for specific binding). Additionally, when the antibody concentration is very high, the sheer number of random collisions between antibodies and the membrane, driven by molecular thermal motion, can lead to non-specific antibody adsorption, even when the “binding energy” of a single non-specific adsorption event (corresponding to -Δ*G*) is very low due to cumulative effects.”

Teacher: “Very well. Then that leaves us with another question: how an extremely high concentration of primary antibody may lead to a not-so-specific signal and an excessive background signal?”

Student (guided): “Principally, high concentration increases collision frequency enough to maximize the site-specific element. Too much concentration will unbalance excess collisions and binding opportunities with other sites with only slightly lower affinity and cause this much higher background. It will also simultaneously dilute the effective concentration of an antibody that can reach the target antigens and may bind to the weakly non-specific sites. The antibody itself tends toward aggregation by promoting this non-specific adsorption.”

Teacher: “From here, what steps seem logically the first steps for troubleshooting? And what is the rationale behind each step from the perspective of Δ*G*, K_D_, concentration, and molecular interactions like K_on_ and K_off_?”

Student (guided): “First, optimize primary antibody concentration-gradient dilutions to find a concentration suitable for achieving specific binding. This concentration provides sufficient negative Δ*G* for Ag-Ab formation with enough molecules participating in the reaction but prevents non-specific binding by reducing non-specific collisions and weakening the total Δ*G* of non-specific binding. In essence, this strategy relies on number adjustment for the reacting molecules, which can balance the kinetics and final equilibrium of specific-non-specific interactions. Second, changing the blocking agents raises the concentration of the current blocking agent and extends the blocking time. This strategy ensures that non-specific binding sites on the membrane are thoroughly occupied, thus rendering conditions thermodynamically very unfavorable (positive values). Third, increasing the number of washes, prolonging individual wash times, or increasing the concentration of detergents in the wash buffer (e.g., Tween-20). In non-specific binding, these complexes are less stable (have larger k_offs_) and their Δ*G* is less negative. Adequate washes will remove free antibodies and provide energy and time for the dissociation of loosely bound non-specific molecules that are in a fast *versus* slow association-dissociation dynamic equilibrium. This strategy can overcome the energy barrier for dissociation and induce thermal motion among molecules.”

### Advantages and challenges of this teaching framework

The primary advantage of this framework is its potential to transform Western blotting instruction from a simple “hands-on” procedural exercise into a rich “minds-on” experience that fosters true “scientific reasoning. Understanding “why” something is done with each experimental procedure (rather than simply memorizing experimental steps) grounds students with a practical understanding of molecular interaction energies (Δ*G*, Δ*H*, Δ*S*) and rates (k_on_, k_off_). By doing so, this paves the way for the development of a rational, systematic troubleshooting mindset, enhancing efficiency and innovation in solving experimental problems, compared with blind trial and error. More importantly, this approach can increase students’ motivation to learn, foster confidence in research, and encourage independent thinking and new problem-solving skills because a coherent explanatory framework is used to traverse an otherwise highly complex experiment.

While integrating thermodynamics and molecular thermal motion principles into the teaching of Western blotting has tangible benefits, certain obstacles arise in their utilization. Firstly, students often have limited knowledge of chemical thermodynamics and kinetics, making it difficult for them to understand the role of biophysical concepts, such as Δ*G*, Δ*H*, Δ*S*, k_on_, and k_off_, in regulating antigen-antibody interactions. The introduction of simplified preparatory materials before the experiment, with the help of metaphors and visualization aids (e.g., animations of molecular motions and binding processes), can lower the cognitive load on students. The learning process can also be more effective by emphasizing conceptual understanding rather than intricate mathematical derivations. Besides, this teaching approach puts higher demands on instructors. In addition to having a strong personal understanding of these principles, instructors should teach the abstract theory associated with experiential knowledge and conduct inquiry-based learning. This point can be facilitated through specific faculty training, the development of full teaching case studies and standardized sets of guiding questions, and encouraging people to share their experiences in this regard. Thirdly, it will put pressure on curriculum design and time allocation to offer experimental training and full and deep biophysical discussion in a limited time. A flipped classroom wherein students learn about theoretical concepts before classes, and then proceed with application and discussion and problem-solving in the classroom may be an optimal strategy in this regard. Alternatively, we can incorporate principle-based teaching into the explanations of each operational step rather than confining it to discrete theoretical instructional modules. Finally, assessing whether students can also apply this strategy flexibly to solve new problems is far more complicated than operational assessment alone. Educators must develop more summative assessment tasks in students’ troubleshooting plan design in which students must provide an extended and in-depth interpretation of the experimental results based on principles rather than through conventional lab reports.

## Conclusion and outlook

Western blotting will remain the main method of detection in the field of immunology and molecular biology. Hence, understanding its underlying principles has become an eminent educational objective for students. The thermodynamic and molecular thermal motion framework explained in this report offers a powerful and comprehensive approach surpassing purely mechanical-operational teaching. By viewing the immunodetection process as a dynamic interplay of molecular forces, affinities, and motion, students can acquire a deeper understanding of more effective troubleshooting, optimal experimental designs, and more critical scientific reasoning.

While the framework presented offers a powerful conceptual tool, future empirical studies will be indispensable for fully assessing its influence on student learning outcomes and their ability to troubleshoot experiments effectively. Future research will therefore incorporate the design and implementation of a quasi-experimental study that compares student cohorts. One cohort would receive traditional instruction and the other would be instructed using our thermodynamic and kinetic framework. This study would implement pre- and post-test components, conceptual inventory questionnaires, and performance-based assessments (e.g., analyzing mock troubleshooting scenarios) to quantify gains in declarative knowledge and practical problem-solving skills. Qualitative input on student engagement and perceived learning value will also be collated through surveys and focus groups.

Looking forward, the fundamental ideas of this framework will find their way into teaching other techniques, such as ELISA, immunoprecipitation, and immunofluorescence, that rely on specific biomolecular recognition. Therefore, we believe that this manuscript, as a well-defined pedagogical proposal, serves as a valuable and immediately applicable resource for educators, providing them with a solid conceptual blueprint to move beyond rote memorization and build a more robust and transferable scientific reasoning ability in their students.
